# Phenotypic characterisation and *ZEB1* mutational analysis in posterior polymorphous corneal dystrophy in a New Zealand population

**Published:** 2009-12-03

**Authors:** Andrea L. Vincent, Rachael L. Niederer, Amanda Richards, Betina Karolyi, Dipika V. Patel, Charles N.J. McGhee

**Affiliations:** 1 Department of Ophthalmology, New Zealand National Eye Centre, Faculty of Medical and Health Science, University of Auckland, Auckland, New Zealand; 2 Ophthalmology Department, Greenlane Clinical Centre, Auckland District Health Board, Auckland, New Zealand

## Abstract

**Purpose:**

Posterior Polymorphous Dystrophy (PPCD) is a genetically heterogeneous corneal dystrophy, with linkage to three different chromosomal loci, with several genes in these loci being implicated. The role of both *VSX1* and *COL8A2* in PPCD remains controversial but recent work suggests that mutations in the transcription factor gene *ZEB1/TCF8* account for disease in up to 30% of subjects, with a significant association with connective tissue abnormalities. This study aimed to determine the phenotype and contribution of *ZEB1* mutations in a New Zealand PPCD population

**Methods:**

Following informed consent, 11 probands with PPCD underwent extensive clinical characterization; including a questionnaire to determine birth history, general health, and the incidence of connective tissue abnormalities, slit lamp examination, photography and in vivo confocal microscopy. Family members were recruited where available. Biological specimens underwent mutational analysis of all nine coding exons of *ZEB1*.

**Results:**

*ZEB1* mutational analysis identified one mutation in the 11 probands (9.1%), a novel mutation in the initiating methionine of exon 1, c.1A→G that results in the protein change p.Met1Val, with resultant aberrant initiation of translation. This mutation segregated with disease in the family, and was not present in 100 control chromosomes. No other *ZEB1* mutations were observed in this cohort.

**Conclusion:**

Recent studies suggest that *ZEB1* mutations may account for PPCD in 18 to 30% of cases, with the majority of the mutations in exons 5 and 7. Clinical and molecular analyses in this New Zealand cohort show a much lower incidence of *ZEB1* sequence change, confirming the genetic heterogeneity of PPCD. We also report identification of a novel mutation in the initiating methionine that removes the Kozak sequence, thereby altering the site of initiation translation.

## Introduction

Posterior Polymorphous Corneal Dystrophy (PPCD) is a frequently asymmetric autosomal dominant corneal dystrophy with characteristic involvement of the Descemet membrane and the endothelium. However, marked phenotypic expressivity and variation is reported and although most affected individuals are often asymptomatic, symptoms may include visual blurring, glare, and rarely the need for penetrating keratoplasty [1]. PPCD has been linked to three chromosomal loci: PPCD1 (OMIM 122000) on chromosome 20p11.2-q11.2, PPCD2 (OMIM 609140) on chromosome 1p34.3 – p32.3, and PPCD3 (OMIM 609141) on chromosome 10p11.2. Putative genes at each locus have been identified but some controversy exists regarding the role of the homeobox gene, *VSX1* in PPCD1. Mutations in this gene were demonstrated segregating with disease in three PPCD families [1,2], but other studies have not replicated these results [3,4], suggesting a different and yet unidentified gene within the PPCD1 locus is responsible for this disease [4-6]. The interval was recently reduced to 10 cM between markers D20S182 and D20S195 [6]. Similarly the *COL8A2* gene within the PPCD2 locus was implicated in this disorder [7], as well as contributing to the pathogenesis of Fuchs’ endothelial corneal dystrophy (FECD). The contribution of this gene has also been called into question as further studies have failed to identify mutations within analyzed PPCD or FECD cohorts [8-10].

Three recent studies investigating a more promising candidate gene at the PPCD3 locus demonstrated disease-causing mutations in the zinc finger E-box binding homeobox 1 gene *ZEB1* (OMIM 189909), previously known as *TCF8* [11-13]. A mutation was confirmed in the original family described by Moroi et al. [14] that linked to this locus [15]. These series also expanded the phenotypic spectrum of PPCD by observing a high incidence of non-ocular connective tissue abnormalities occurring in association with *ZEB1* mutations and PPCD, predominantly inguinal and abdominal hernias.

Marked corneal phenotypic heterogeneity in PPCD exists with the clinical spectrum manifesting as nodular, vesicular, or blister like lesions within the endothelium, which may be isolated, cluster, or form curvilinear tracks - a band or “railroad track” appearance delineated by strips of condensation and diffuse irregularities in the Descemet membrane, and a more diffuse type with variable amounts of grey tissue and irregularity at the level of the Descemet membrane. Peripheral iridocorneal adhesions and elevated intraocular pressure are also observed [16,17].

The characteristic histological features demonstrate the transformation of the endothelial cell phenotype to an epithelial-like cell with desmosomes, stratification, layering, and microvilli [18] but as the majority of cases do not require surgical intervention, tissue specimens are rarely available for histological analysis. However, the advent of imaging modalities such as in vivo confocal microscopy (IVCM) permit comprehensive characterization of these vesicular or linear deposits [19-22], enabling clarification of the phenotype in situ. This is particularly useful for very mild disease, and in the absence of a family history.

In this study we aimed to characterize a cohort of New Zealand patients with PPCD as extensively as possible, from both an ocular and systemic perspective, and undertake mutational analysis of the *ZEB1* gene.

## Methods

Eleven (n=11) probands with a diagnosis of PPCD, were recruited from the Ophthalmology Department of Auckland District Health Board over a 12 month period and reviewed in the University Clinic, Dept of Ophthalmology, University of Auckland. Family members were also recruited where available. The study design adhered to the tenets of the Declaration of Helsinki with Institutional Research Ethics Board approval (NTX/06/12/161), and all patients provided informed consent before being entered into this study.

### Questionnaire

All subjects completed a general health questionnaire which included specific questions based on associations of PPCD described in earlier publications [11-13], specifically regarding the presence of specific soft tissue abnormalities (hernia, hydrocoele, Dupuytren’s contracture, bony lumps, Osgood-Schlatter disease, and spinal changes), as well as hearing problems, otosclerosis, and kidney disease.

### Clinical

All subjects underwent extensive clinical examination including Snellen visual acuity, auto-refraction, corneal topography, and pachymetry using a combined Placido/slit-scanning elevation tomography system (Orbscan II; Bausch & Lomb Surgical, Rochester, NY), slit lamp examination and photography, and laser scanning in vivo confocal microscopy (IVCM) using the HRTII (Heidelberg Retina Tomograph II, Rostock Corneal Module (RCM) (Heidelberg Engineering GmbH, Heidelberg, Germany). Peripheral venous blood samples were obtained for DNA extraction.

### Molecular

DNA extraction from blood used the salt extraction method [23]. PCR amplification of all nine *ZEB1* exons and flanking intronic regions was undertaken in all samples using previously described primers and conditions (Exon 1 [11], Exons 2-9 [12]). Following column purification with the HighPure PCR purification kit (Roche Diagnostic, Mannheim, Germany), product was sequenced directly according to protocols accompanying the ABI BigDye Terminator kit v3.1. Bidirectional sequencing of amplicons was undertaken on an ABI 3100 prism genetic analyzer (Applied Biosystems Inc, Foster City, CA). Nucleotide sequences were compared manually and with CodonCode Aligner v.2.0.6 (CodonCode, Dedham, MA) against the published *ZEB1* genomic DNA (GenBank NC_000010.9), and complementary DNA (cDNA) reference sequence (NM_030751.4). This variant (2) uses an alternate in-frame exon, compared to variant 1, resulting in a longer protein (isoform b) with a distinct NH_2_-terminus, compared to isoform a. When a sequence variant was confirmed, family members were tested to determine segregation with the disease.

The exon 1 sequence variant was screened for by NLAIII restriction enzyme digest in 50 unaffected control individuals (100 control chromosomes) using the following Digest mix: 9 μl PCR product, 0.2 μl NLAII enzyme, 0.12 μl BSA, 1.2 μl NE buffer 4, and 1.48 μl sterile water. This was digested overnight at 37 °C, 2ul BlueJuice^TM^ (Invitrogen, Carlsbad, CA) added to each reaction tube, and run on a 3% agarose gel.

## Results

**Figure 1:**
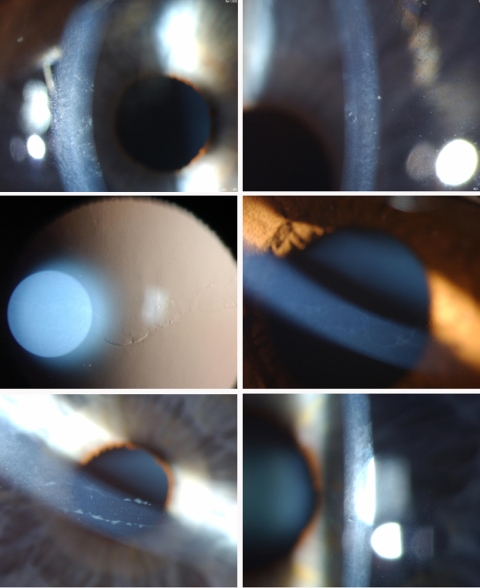
Slit lamp photographs of three corneas demonstrating variation in corneal phenotype in PPCD Top row: R and L cornea of Patient #5, scattered vesicular endothelial lesions Middle row: Patient #2 Retroillumination and direct slit lamp of a band lesion, with a gray halo surrounding lesion Bottom row: Band or railroad track phenotype in Patient #7, with thickened Descemet’s membrane at the edges of the band. LE shows small isolated vesicular lesions.

**Figure 2:**
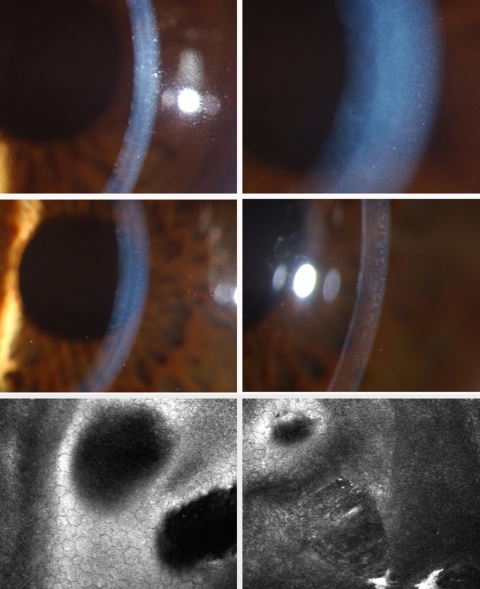
Phenotype of family members with *ZEB1* p.1Met→Val mutation: Upper Row: Daughter (Patient #6a) age 12: slit lamp images of cornea with prominent nerves, diffuse haze. Middle Row: Father (Patient #6) age 45 more diffuse clouding with isolated vesicles and prominent corneal nerves. Lower Row: father (Patient #6) IVCM.

Eleven probands were recruited; their demographics and clinical details are listed in Table 1. Four additional affected family members were identified, such that the cohort included four familial and seven sporadic cases. Three of the individuals were of Polynesian ancestry. (New Zealand Maori n=1, Samoan n=1, Tongan n=1) All of the cases were determined to be PPCD on the basis of family history when present, bilaterality, exclusion of birth trauma or forceps delivery, and typical features observed on slit-lamp and IVCM. Clinical appearances of corneas and variable phenotypes are demonstrated in Figure 1 and Figure 2.

**Table 1:** Demographic and ocular data for eleven subjects with PPCD.

**Patient number**	**Sex**	**Age**	**Ancestry**	**RE BCVA**	**LE BCVA**	**Orbscan II topography CCT**		**Orbscan II topography thinnest**		**Endothelial cell density**		**Family history**
						**RE**	**LE**	**RE**	**LE**			
1	M	37	Caucasian	6/6	6/6	495	482	488	479	1650	x	Y - CDB
2	M	10	NZ Maori	6/6-	6/7.5	511	562	506	539	x	2358.3	Y
3	M	48	Polynesian	6/3.8	6/120	x	530	x	524	x	1783.33	N
4	F	80	Caucasian	6/9	6/9	x	x	x	x	816.667	x	N
5	M	57	Caucasian	6/3.8	6/3.8	558	x	550	x	1225	883.33	NK
6	M	45	Caucasian	6/6	6/15	594	631	583	623	1466.67	1991.67	Y
6a	F	12	Caucasian	6/18	6/18	646	638	637	628	1133.333	x	Y
7	F	51	Caucasian	6/4.8	6/4.8	628	608	611	591	1191.667	2716.67	N
8	F	18	Caucasian	6/15	6/6	534	517	527	508	1366.67	x	Y
9	F	56	Caucasian	6/3.8	6/6	553	553	536	542	2100	1233.33	N
10	F	34	Caucasian	66/4.8	6/9	x	x	x	x	2800	1291.67	N
11	M	32	Tongan	6/6	6/7.5	x	x	x	x	x	x	NK

In the table, M = male and F = female, Age is in years, RE = Right eye, LE = left eye, BCVA = best corrected visual acuity, CCT = central corneal thickness, measured in microns, x = information not recordable, or not obtained. Y = yes, N = no, NK = not known, CDB = corneal dystrophy of Bowman Layer. Comment patient #3; LE had penetrating keratoplasty following blunt trauma, PPD in RE.

**Figure 3:**
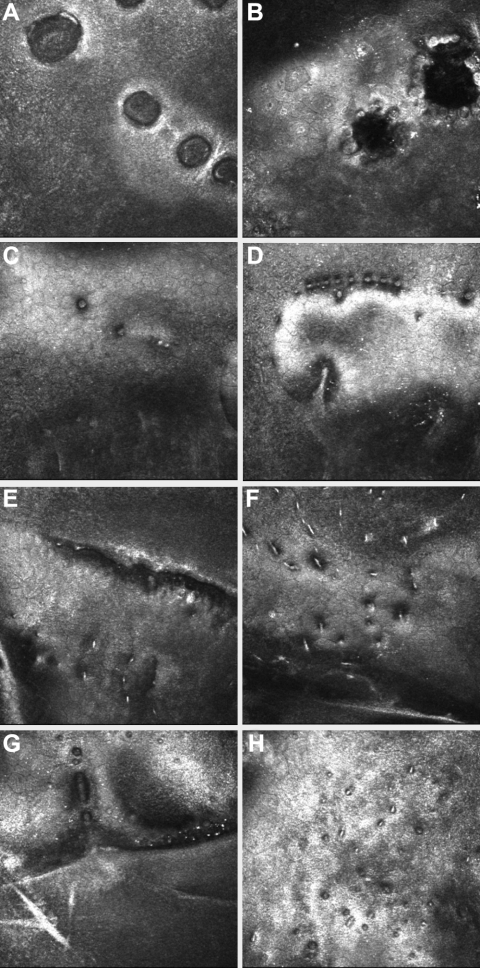
IVCM images of phenotypes of PPCD. Vesicular type: **A**, **B** (Patient #5) and **C**, **D** (Patient #1) - vesicular type. **A** is at the level of DM, showing a row of focal elliptical vesicular lesions, each has a hyper-reflective halo. **B** is at the level of the endothelium. Vesicular lesions have hypo-reflective centers and scalloped borders. **C**: multiple small focal vesicular endothelial lesions. Band Type **E**, **F** (Patient #7) and **G**, **H** (Patient #4). **E** shows the edge of a band lesion. It demonstrates the undulation of DM and endothelial surface. **F**: this image from within a band demonstrates abnormal dimpling of endothelial cells. **G** shows the edge of a band lesion. It demonstrates the undulation of DM and endothelial surface and hyper-reflective DM. **H**: within the band lesion. Abnormal dimpling of endothelial cells with hyper-reflectivity of the overlying Descemet's membrane. (Each image is 400 µm×400 µm.)

IVCM in all patients demonstrated abnormal endothelial appearances (Figure 2 and Figure 3). Vesicular PPCD lesions appeared as focal circular or elliptical regions with hypo-reflective centers and scalloped borders. These lesions were often surrounded by hyper-reflective “halos” in the overlying Descemet’s membrane, correlating with the “grey halos” observed on slit-lamp biomicroscopy. Band lesions exhibited well defined borders with abnormal dimpling of endothelial cells within the band and hyper-reflectivity of overlying Descemet’s membrane. Undulation of the Descemet membrane and the endothelial surface were observed in the region of PPCD lesions.

**Figure 4:**
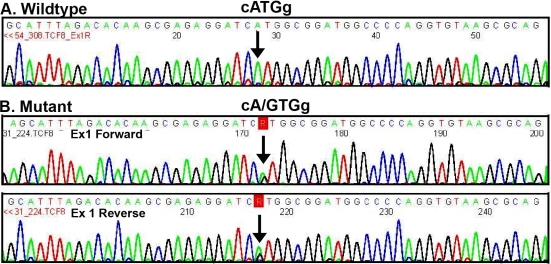
Electropherograms demonstrating *ZEB1* p.1Met>Val mutation in exon 1. **A**: Wild-type sequence, **B**: Mutant heterozygote with c.1A>G demonstrated with bidirectional sequencing.

Bidirectional sequencing of *ZEB1* identified non-pathogenic sequence variations, and one probable disease-causing novel sequence variation c.1A→G which results in the protein change p.1Met→Val. (Figure 4; electropherogram) This change segregated with disease in the family, and was not observed in 100 control chromosomes. Other non pathogenic sequence variations/single nucleotide polymorphisms (SNPs) detected have been previously described and are listed in Table 2.

**Table 2:** SNPs and mutation table.

**Patient ID**	**Pathogenic variant**	**Non-pathogenic sequence variants**	
	**Exon 1**	**Exon 3**	**Exon 6**
1		c.260-538C>T, het	c.685-15G>A, hom
2		c.260-154A>G, het	c.685-15G>A, het
3			c.685-15G>A, hom
4		c.260-154A>G, het	c.685-15G>A, hom
5			c.685-15G>A, het
6	c.1A>G het, p.Met1Val		c.685-15G>A, hom
6a	c.1A>G het, p.Met1Val		c.685-15G>A, hom
7			c.685-15G>A, het
8			c.685-15G>A, hom
9			c.685-15G>A, het
10			c.685-15G>A, hom
11		c.260-154A>G, het	c.685-15G>A, hom

No sequence variant detected in exons 2,4,5,7-9. In the table, “het” represents heterozygous and “hom” represents homozygous sequence variant.

**Figure 5:**
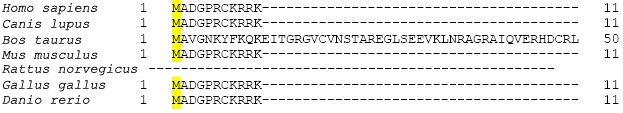
Homology modeling demonstrates conservation of the initiating methionine in seven vertebrate species.

Prediction of potential coding fragment in the reference mRNA (NM_0030751.4) sequence containing the c.1A→G was performed using the BESTORF program (Softberry.Inc, Mount Kisco, NY), and predicted translation initiation at nucleotide 788 which encodes the methionine 219 in exon 6, resulting in the normal 1124 amino acid protein being truncated by 218 amino acids at the COOH-terminus. Homology modeling shows this initiating methionine is highly conserved in seven vertebrate species (Figure 5).

## Discussion

The zinc E-finger homeodomain binding box protein *ZEB*1 located on chromosome 10p11 was previously known as transcription factor 8 (*TCF8*) as it represses the T-lymphocyte-specific IL2 gene (OMIM 147680) expression [24]. This transcription factor plays a critical role in embryonic development, specifically in the regulation of type I collagen expression and in the repression of the epithelial phenotype, which is critical for the maintenance of an endothelial phenotype.

One of the major morphologic abnormalities in PPCD is the transformation of the endothelium into cells with epithelial characteristics. Ultrastructurally, this appears as focal loss and degeneration of the endothelial cell monolayer, several layers of stratified squamous cells containing tonofilaments, desmosomal attachments and cytokeratin, as well as microvilli projecting into the anterior chamber [18]. Immunohistochemical analysis of this abnormal posterior corneal surface with cytokeratin staining shows a predominance of CK7 and CK19 – epithelial cell markers usually present in ductal and glandular epithelia [25].

Descemet’s membrane (DM) also demonstrates abnormal lamination and abnormal deposition of fibrillar and basement membrane collagenous material in its posterior portion, with irregular thickenings or excrescences, similar to guttata [18]. However, it has been proposed that the change in Descemet in PPCD is a secondary response to a stressed endothelium [16].

A complex binding site for ZEB1 protein exists in the promoter of a collagen gene, *COL4A3,* which is linked to the autosomal recessive form of Alport syndrome [12]. Notably, type IV collagen is a major component of basement membranes.

For mutational analysis of any gene in a given disease to be meaningful, the phenotype should be well-defined, in an effort to avoid phenocopies. This may be troublesome when considerable variability in phenotypic expression exists, as in PPCD. All of our probands had bilateral disease, with the characteristic vesicular, band, or diffuse appearance at the level of the endothelium, frequently associated with a gray halo surrounding the lesions at the level of DM. The IVCM changes observed are consistent with those previously described [19-22]. A differential diagnosis of PPCD includes birth trauma or tears in DM from forceps delivery or Haab’s striae in congenital glaucoma. We excluded traumatic birth history or forceps delivery in all our probands, although this may be subject to recall bias. Differentiation between these entities can also well delineated by clinical appearance, progression and associations, histopathologically, and by IVCM [26-28].

In delivery forceps related injury, a hypertrophic ridge of the Descemet membrane may be identified by IVCM [28]. In Haab’s striae there is usually associated megalocornea, and on IVCM the striae appear as highly reflective acellular scar tissue with undefined borders located anterior to the endothelial layer, extending toward the posterior stroma. In addition, the mean keratocyte density of the posterior stroma is reduced, and stromal nerve fibers show an abnormal morphology of convoluted “coil-shaped” nerves [27].

An absence of family history is not uncommon in PPCD, and has previously been demonstrated in association with *ZEB1* mutations [11]. This may be partly attributed to lack of ocular symptoms and mild clinical phenotypes potentially being overlooked on examination, however, nonpenetrance is also reported in *ZEB1* associated PPCD [12,13], therefore where possible thorough genotyping and phenotyping should be performed on all relatives.

An overlap with other clinical phenotypes and PPCD exists, in particular with FECD, the association being strengthened by early reports reporting mutations in the *COL8A2* both in PPCD and FECD, suggesting a similar etiology [7]. The authors suggested that underlying disturbance of the role of type VIII collagen in influencing the terminal differentiation of the neural crest derived corneal endothelial cell contributed to these disorders. In addition, these diseases share features of endothelial metaplasia and secretion of an abnormal Descemet membrane in the form of a posterior collagenous layer with a small or absent posterior non-banded zone, which contains Type VIII collagen - normally only seen in the anterior banded component [29]. Indeed, ocular characteristics related to PPCD have been reported in relatives of patients with FECD and vice versa [30]. Mutational analysis of *ZEB1* in a Chinese population with late onset FECD identified one mutation, suggesting it is not a major contributor to FECD [30].

The frequency of *ZEB1* mutations in PPCD cohorts has varied considerably amongst the reports available to date, and these frequencies are summarized in Table 3. Initial studies had small cohorts with high mutational frequencies (40-45%) [12,13]. The largest published series to date, demonstrated eight unique mutations (25%) in 32 probands [11], and one other study similarly demonstrated mutations in 5/29 =17.3% PPCD [31]. The low frequency observed in our cohort is predominantly explained by the sample size, but yet again highlights the genetic heterogeneity of this corneal disorder. Founder effect is unlikely to be a contributory factor however as all the mutations identified to date have been unique. Notably, the ethnic mix of our population varies from the Caucasian cohorts reported, specifically with the inclusion of three Polynesian individuals (NZ Maori, Tongan, and Samoan).

**Table 3:** Reported studies of TCF8/ZEB1 mutational analysis in PPCD, including current study.

**Authors**	**Sample size**	**Mutations**	**%**	**Hernias**
Aldave et al. [11]	32	8	25	8/8
Krafchak et al. [12]	11	5	45	10/11
Liskova et al. [13]	10	4	40	0/10
Shah et al. [31]	29	5	17.9	Not reported
This article	11	1	9.1	1/11
Total	93	24	25.8	47.5%

The observation of a non-ocular phenotype associated with PPCD manifesting as inguinal hernias/ hydroceles/ bony abnormalities has been replicated [11,12] as has the observation of nonpenetrance [12,13] and mutations present in isolated, apparently non-familial cases [11,12]. Only one inguinal hernia was present in our cohort, and did not occur in conjunction with *ZEB1* disease.

All of the mutations described to date are unique; many are frameshift and nonsense with some missense mutations - the majority occurring in the largest exon, (Exon 7) which contains the homeodomain and five zinc finger domains. The c.1A→G missense transition observed in the reported cohort results in the protein change p.1Met→Val. Two previous mutations involving the initiating methionine are reported –p.Met1Arg , c.2T→G) [11] and p.Met1Thr, c.2T→C [31]. This is the only amino acid residue where numerous mutations are reported, suggesting this may be a “hot spot”, as is observed with the 124 and 555 arginine in *TGFBI -* associated corneal dystrophies [32]. The clinical phenotype segregating with the p.Met1Arg and p.Met1Thr mutations was however not clearly characterized, so it is not clear if the p.1Met→Val phenotype is similar or different to these entities.

The predicted effects of this mutation will result in a COOH-terminal truncated protein, as translation will likely be initiated further downstream. Initiation of translation by the 40S ribosomal subunits does not begin at the first ATG (AUG of mRNA) observed, but rather the optimal ATG initiating codon and flanking sequence, known as a Kozak sequence. A purine at position -3 and a guanine at position +4 contribute significantly to enhance the translation efficiency [33-35]. This optimal context has been determined to be CCNCCAUG. The first disease described with a mutation in the Kozak sequence was Beta-thalassemia [36] and subsequently it has been identified in a range of disorders including Peters anomaly [37], low bone density [38], and Graves’ disease [39]. Prediction of the next Kozak sequence, using BESTORF program suggests the resultant protein would be 906 amino acids long and commence at the nucleotide 788 (wild-type AGGATCATGG with subsequent production of a CGTCACATGA). Whether this truncated protein is functional has not been determined.

This series of patients not only describes a novel mutation within *ZEB1* in PPCD, but is unique in several other ways. It also highlights the genetic heterogeneity of PPCD. Although the individual series reported are too small to accurately assess prevalence, it is possible that population specific variances in *ZEB1*-related PPCD exists.

This is the first of the published studies that clearly demonstrates in vivo confocal microscopy observations in all of the patients with PPCD, thereby excluding other similar, potentially confusing pathologies, such as corneal birth-forceps injury. This level of phenotypic scrutiny has not been described before in association with mutational analysis. Similarly this PPCD cohort was specifically questioned about non-ocular phenotypes, whereas this was not recorded in the Shah series [31]. Key corneal characteristics, such as endothelial count and pachymetry, have not been routinely included in previous publications and relatively brief descriptions of the corneal phenotypes segregating with the TCF8 mutations are described in two major studies [11,13], and the third concludes that “the PPCD3 ocular phenotype resulting from TCF8 mutation appears to be a simple PPCD phenotype, although it shows great variation in range of severity” [12]. Unfortunately, in this latter study, no images are provided; there are no IVCM data, and there are no details regarding variation in endothelial count or corneal thickness.

The published data thus far on the genetic contributions of *ZEB1*, in the context of our knowledge of *VSX1* and *COL8A2*, suggest and highlight that PPCD represents a complex disease with a great amount of genetic and phenotypic heterogeneity. Although there appears to be a strong association with a non-ocular phenotype with *ZEB1* mutations, this was not obvious in our cohort. Similarly the *ZEB1* ocular phenotype does not appear to be distinctive to allow it to be delineated clinically from the other forms of PPCD. On-going genotyping and development of enhanced phenotyping tools may allow this distinction to be better clarified.

Analysis of *ZEB1* in this cohort of PPCD patients confirms the pathogenicity of this gene but suggests the frequency differs between populations. Combining the 5 series to date, including this series, suggests *ZEB1* mutations in PPCD probably accounts for 25% of the disease. Three of the reported mutations to date including the novel Met1Thr in this cohort involve the initiating methionine, suggesting this site is a potential hot spot. *ZEB1* therefore is one of the more significant genes identified to date in the pathogenesis of this disease.
